# Missing a Phage: Unraveling Tripartite Symbioses within the Human Gut

**DOI:** 10.1128/mSystems.00105-19

**Published:** 2019-05-07

**Authors:** Jeremy J. Barr

**Affiliations:** a School of Biological Sciences, Monash University, Clayton, Victoria, Australia

**Keywords:** bacteriophage, symbioses, gut, microbiome

## Abstract

Tripartite symbioses between bacteriophages, the epithelial cell layers of the human gut, and bacterial symbionts may play an important and unrecognized role in the function of the gut microbiome. Traditionally, phages residing within the gut were considered to interact only with their bacterial hosts and thereby to facilitate indirect interactions with the epithelial cell layers, and yet a growing body of literature is demonstrating the surprising and diverse ways in which phages directly interact with the eukaryotic cells, organs, and systems of the body. Phages can adhere to mucosal surfaces, bind and transcytose epithelial cells, and deliver proteins and nucleic acids to eukaryotic cells directly. These interactions could establish positive-feedback loops leading to the selection of bacterial hosts and their phage symbionts in the gut. The members of my laboratory are working to expand our knowledge on the phage-eukaryote interactions and to redefine the concept of tripartite symbioses within the human body.

## Abstract (precis)

Tripartite symbioses between bacteriophages, the epithelial cell layers of the human gut, and bacterial symbionts may play an important and unrecognized role in the function of the gut microbiome. Traditionally, phages residing within the gut were considered to interact only with their bacterial hosts and thereby to facilitate indirect interactions with the epithelial cell layers, and yet a growing body of literature is demonstrating the surprising and diverse ways in which phages directly interact with the eukaryotic cells, organs, and systems of the body.

## PERSPECTIVE

The human body is colonized by a diverse collective of microorganisms, including bacteria, fungi, protozoa, and viruses (1, 2). The large intestine harbors the body’s most densely populated microbial ecosystem, with an estimated 10^13^ to 10^14^ microbial cells per gram of fecal matter (1). It is well established that our gut microbial flora has coevolved with us, forming “symbiotic relationships” with our bodies that are largely responsible for our overall well-being (2). The smallest entities within the gut microbial community are the bacterial viruses—bacteriophages, or “phages” for short (3). Phages constitute essential components of our gut microbiome; carrying a rich repertoire of genes and exerting strong selective pressures on their bacterial hosts (4, 5). Our bodies are frequently and continuously exposed to high numbers of phages, and we secrete several billion per gram of feces. Despite their high numbers in the body, phages cannot infect human cells in the same way that they infect their bacterial hosts. The cell surface receptors and intracellular machinery of human and bacterial cells are simply too different. A consequence of this lack of tropism has been the assumption that phages simply do not interact with eukaryotic cells at all, and yet this assumption is inherently wrong, and a growing body of literature is demonstrating the surprising and diverse ways by which phages directly interact with eukaryotic cell layers, organs, and systems (6–11). In this perspective, I summate the classical linear symbioses between phage and bacterium and between bacterium and animal host and then redefine the concept of tripartite symbioses within the context of the human body and describe how the research program in my laboratory will shape this burgeoning new field of microbiology.

## LINEAR SYMBIOSES BETWEEN PHAGE, BACTERIUM, AND ANIMAL HOSTS

The bacteriophage was defined by Félix d'Hérelle in 1917 as “a particle which proliferates at the expense of bacteria; and as a result, is capable of assimilation and is indefinitely cultivable in series *in vitro*.” Initially, the symbioses between virulent phages and their bacterial hosts were considered chiefly antagonistic in nature. As such, virulent phages were used as model organisms to describe population dynamics, including frequency-dependent selection, and as drivers of coevolutionary processes. Knowledge of symbioses between phage and bacterium was further extended with the discovery of lysogeny, whereby temperate phages may incorporate their genomes into the host bacterium cell(s) as a prophage. Temperate phages influence their bacterial host via dynamic mechanisms, including protection from lysis by similar phages via superinfection exclusion, and carry a rich genetic repertoire that can affect bacterial physiology through lysogenic conversion and facilitate horizontal gene transfer.

**FIG 1:**
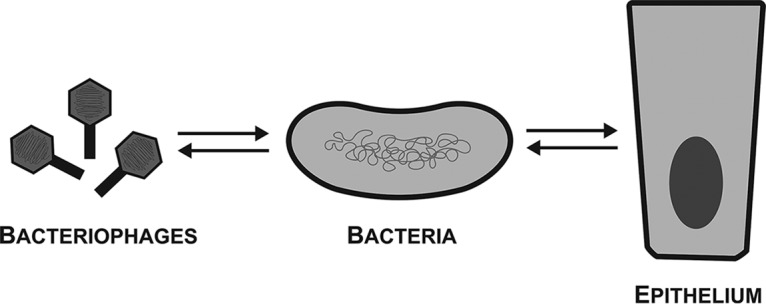
The linear symbiosis concept encompasses phage–bacterium and bacterium–epithelium interactions and symbioses. Bacteriophages form symbioses with their bacterial hosts, including lysis, lysogeny, and horizontal gene transfer mechanisms. Bacteria engage in diverse symbioses with epithelial cells, organs, and systems of their animal hosts, which includes the provision of novel metabolic functions, modulating signaling pathways, and pathogenesis. Within this linear symbiosis framework, bacteriophages may interact with and influence their animal hosts only through indirect effects.

Within the context of an animal host, there are equally dynamic symbioses occurring between bacteria and the epithelial cells, organs, and systems of the animal host (12). Commensal bacteria can form mutualistic symbioses with their animal host through provision of novel metabolic capabilities and can even modulate eukaryotic signaling pathways that regulate animal host behavior, development, and growth. However, these mutualisms can break down, with commensals transitioning into the bacterial pathogens known to cause infections and disease (12). The linear symbiosis concept encompasses phage-bacterium and bacterium-epithelium interactions and symbioses and the ability of phages to influence their animal host via indirect effects (Fig. 1). And yet this concept breaks down in considering recent research demonstrating direct interactions and symbioses between phages and their animal hosts. My research program aims to define new mechanisms of phage-eukaryote interactions and to establish a new model of tripartite symbioses within the human gut.

## MECHANISMS FOR PHAGE-EUKARYOTE INTERACTIONS

It is well accepted that phages cannot infect human or eukaryotic cells in the same way that they infect their bacterial hosts. However, a recent wave of research has highlighted the diverse and surprising ways in which phages can interact with and influence human cells and those of other higher vertebrates (6–11). During my postdoctoral work, my colleagues and I discovered a novel symbiotic relationship between phages and eukaryotic hosts, termed the bacteriophage adherence to mucus—or BAM—model, whereby phages adhering to mucosal surfaces provided a previously unrecognized antimicrobial defense (6, 7). We demonstrated that T4 phage was enriched in mucus layers via binding interactions between mucin glycoproteins and immunoglobulin-like domains exposed on phage capsids. The immunoglobulin protein fold is notable in that it can support a high degree of variation (>10^13^ potential mutations) while still maintaining the structural stability of the protein fold. Within the human gut, phage-encoded immunoglobulin-like domains were targeted for hypervariation via reverse transcriptase based mechanisms, allowing rapid evolution and adaptation of these domains within the gut (13). On this basis, we speculated that phages utilize the variable immunoglobulin-like protein fold to adapt their mucus adherence across diverse mucosal surfaces along the human gut and across other mucosal layers and animal hosts. The members of my laboratory are combining experimental evolution with gut-on-chip devices to address whether phage adaptation of immunoglobulin-like domains facilitates increased adherence and antimicrobial protection of mucus layers. This work will build upon concepts of coevolution to include tripartite symbioses between phages, the mucosal surfaces of the human gut, and the bacterial symbionts residing within the mucus layer.

Once past the mucosal layer, phages inevitably contact and interact with the eukaryotic epithelial cell surface. Although incapable of infecting eukaryotic cells, naturally occurring phages have been demonstrated to bind eukaryotic cellular receptors directly (10). Once bound, phages can be endocytosed by epithelial cells and trafficked throughout the endomembrane system and phage proteins and nucleic acids can be released into the eukaryotic cell (8). This process has been employed in the field of nanotechnology, where phages are engineered to display ligands on their capsid that target and bind specific eukaryotic receptors, for use as viral gene delivery vectors and nano-carriers (14). Phage capsids can be packaged with recombinant DNA, RNA, and proteins, allowing the targeted delivery of genes and enzymes into human cells of interest. Through these mechanisms, phages are capable of transducing eukaryotic cells, with phage-carried genetic material being delivered, transcribed, and translated into functional proteins by the eukaryotic cellular machinery (15), by as-yet-unknown mechanisms.

The members of my laboratory recently proposed a broadly applicable mechanism for the direct interaction of naturally occurring phages with epithelial cell layers (8). *In vitro* studies demonstrated that human cell layers from the gut, lung, liver, kidney, and brain were able to take up and traffic phages. Once internalized, phages were transported throughout all compartments of the eukaryotic cell, with a proportion of these phages eventually secreted on the opposing side of the cell layer. Extrapolating from these results, we estimated that, per day, the average human body absorbs 3.1 × 10^10^ phages from the gut, transporting these phages across the gut epithelial cell layers and depositing them into the lymphatic and circulatory systems of the body (8). Continuing with this research theme, the members of my laboratory are addressing issues concerning how phages enter, traffic, and deliver nucleic acids into the eukaryotic cells, which innate immune pathways are activated upon phage transcytosis, and whether naturally occurring phages transduce epithelial cell layers to mediate novel tripartite symbioses within the gut environment. This research challenges biomedical dogma and suggests that phages do directly interact with human cells in diverse and unexplored ways.

## MODEL OF TRIPARTITE SYMBIOSES

**FIG 2:**
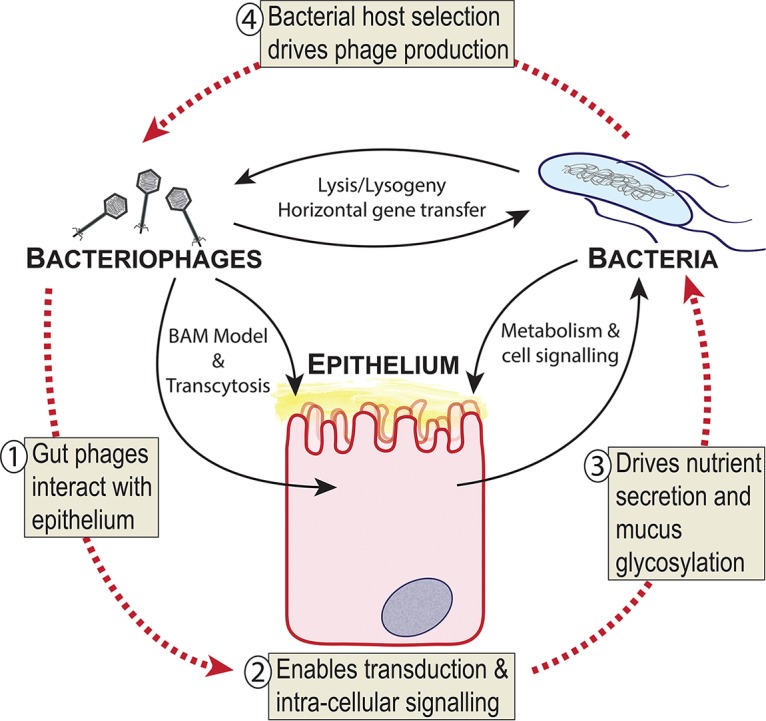
Model of tripartite symbioses between bacteriophages, epithelium, and bacteria within the gut. (Step 1) Gut phages are known to interact directly with epithelial cells through adherence to mucus (BAM model), binding of cellular receptors, and transcytosis (6–8, 10). (Step 2) Phage-epithelium uptake enables delivery of phage proteins and nucleic acids that allows transduction and activation of intracellular signaling (11, 14, 15). (Step 3) Phage-mediated effects may drive nutrient secretion or mucus glycosylation back into the gut environment to select for specific bacterial symbionts. (Step 4) Positive selection of bacterial hosts drives further phage production in the gut environment.

My research program aims to establish new mechanisms of phage-eukaryote interactions and incorporate these into a model of tripartite symbioses within the human gut (Fig. 2). Based on prior research (6–11, 14, 15), this model proposes that naturally occurring phages directly interact with the epithelial cells of the gut, forming tripartite symbioses that can modulate eukaryotic responses and feedback loops that influence the bacterial microbiome. Through BAM mechanisms, gut phage populations modulate bacterial communities residing within mucosal layers, driving strain diversification and long-term adaptation (6, 7). Past the mucus layer, the internalization and transcytosis of phages allow their direct interaction with the eukaryotic cell, potentially activating signal transduction and innate immunity and facilitating gene delivery and transduction (3, 8, 10, 11, 15). An important issue that the members of my laboratory are addressing is whether these mechanisms occur naturally within the human gut. If so, these tripartite symbioses could have far-reaching implications for the function of the gut microbiome. Phage modulation of the epithelial cell responses could regulate the production and glycosylation of the mucus layer, dampen or activate inflammatory immune responses, and coordinate the secretion of the key metabolites that serve as nutrient sources for many gut bacterial symbionts. These phage-driven interactions could establish positive-feedback loops between the epithelial cell layer and the bacterial microbiome, ultimately selecting for—or against—potential bacterial hosts for the phage symbionts, forming tripartite symbioses among the phage, epithelium, and bacterium (Fig. 2). Although the symbiotic roles of phages within the human gut remain largely unexplored, I expect research over the coming years to demonstrate that phages interact with the cells, organs, and systems of the human body as often, and in ways as diverse, as bacterial symbionts.
